# Individualized chiropractic and integrative care for low back pain: the design of a randomized clinical trial using a mixed-methods approach

**DOI:** 10.1186/1745-6215-11-24

**Published:** 2010-03-08

**Authors:** Kristine K Westrom, Michele J Maiers, Roni L Evans, Gert Bronfort

**Affiliations:** 1 Northwestern Health Sciences University, Wolfe-Harris Center for Clinical Studies, 2501 West 84th Street, Bloomington, MN 55431, USA

## Abstract

**Background:**

Low back pain (LBP) is a prevalent and costly condition in the United States. Evidence suggests there is no one treatment which is best for all patients, but instead several viable treatment options. Additionally, multidisciplinary management of LBP may be more effective than monodisciplinary care. An integrative model that includes both complementary and alternative medicine (CAM) and conventional therapies, while also incorporating patient choice, has yet to be tested for chronic LBP.

The primary aim of this study is to determine the relative clinical effectiveness of 1) monodisciplinary chiropractic care and 2) multidisciplinary integrative care in 200 adults with non-acute LBP, in both the short-term (after 12 weeks) and long-term (after 52 weeks). The primary outcome measure is patient-rated back pain. Secondary aims compare the treatment approaches in terms of frequency of symptoms, low back disability, fear avoidance, self-efficacy, general health status, improvement, satisfaction, work loss, medication use, lumbar dynamic motion, and torso muscle endurance. Patients' and providers' perceptions of treatment will be described using qualitative methods, and cost-effectiveness and cost utility will be assessed.

**Methods and Design:**

This paper describes the design of a randomized clinical trial (RCT), with cost-effectiveness and qualitative studies conducted alongside the RCT. Two hundred participants ages 18 and older are being recruited and randomized to one of two 12-week treatment interventions. Patient-rated outcome measures are collected via self-report questionnaires at baseline, and at 4, 12, 26, and 52 weeks post-randomization. Objective outcome measures are assessed at baseline and 12 weeks by examiners blinded to treatment assignment. Health care cost data is collected by self-report questionnaires and treatment records during the intervention phase and by monthly phone interviews thereafter. Qualitative interviews, using a semi-structured format, are conducted with patients at the end of the 12-week treatment period and also with providers at the end of the trial.

**Discussion:**

This mixed-methods randomized clinical trial assesses clinical effectiveness, cost-effectiveness, and patients' and providers' perceptions of care, in treating non-acute LBP through evidence-based individualized care delivered by monodisciplinary or multidisciplinary care teams.

**Trial registration:**

ClinicalTrials.gov NCT00567333

## Background

It is well recognized that low back pain (LBP) is one of the most prevalent and costly problems facing the US health-care system [1-3]. Approximately 80% of individuals will experience non-specific LBP in their lifetime[4] and 75% will experience lingering problems one year after onset[5]. In the United Kingdom, the incidence of chronic low back disability rose exponentially for two decades through 1994,[6] and for some patients the associated psychological distress and illness behaviors become as disabling as the LBP itself[7]. In the United States, the costs attributable to LBP continue to soar and are estimated to exceed $100 billion dollars annually[8].

At present, more than 500 randomized controlled trials have been published evaluating conservative and alternative treatments for LBP. From the results of these trials, there appears to be no one treatment for non-specific LBP that is best for all patients, but instead, several viable treatment options [9-12].

Although LBP is common, each patient and their low back pain experience is unique. Using biopsychosocial measures to individualize treatment may be more appropriate than a "one size fits all" approach. Having multiple efficacious treatments also introduces greater opportunity for choice, which, by allowing expression of patient preference, may positively affect treatment outcomes[13]. Additionally, by combining the efforts and care of multiple providers, it is hypothesized that a collective approach can exceed what can be accomplished by monodisciplinary care, particularly for chronic conditions [14-18].

There is evidence from at least one RCT[19] to suggest that providing individualized treatment within a multidisciplinary, conventional medicine care pathway results in faster return to work for chronic LBP patients. Integrative care, including both CAM and conventional therapies for chronic LBP, is an approach with the potential to improve upon the fragmented and extremely costly delivery system currently in place and is worthy of further rigorous exploration.

This paper describes the protocol of a mixed-methods randomized clinical trial designed to compare monodisciplinary, chiropractic care with multidisciplinary, integrative care for chronic LBP. Both the monodisciplinary and multidisciplinary care are delivered within the context of individualized, evidence-based clinical care pathways.

### Primary Aim

The primary aim of the study is to determine the relative clinical efficacy of 1) **monodisciplinary, chiropractic care **and 2) **multidisciplinary, integrative care **for LBP of greater than 6 weeks duration, in both the short-term (after 12 weeks) and long-term (after 52 weeks). Patient-rated back pain is the primary outcome measure.

### Secondary Aims

The secondary aims of the study are to determine the short- and long-term relative efficacy of the two interventions using the following secondary patient-rated outcome measures: frequency of symptoms, low back disability, fear avoidance, self-efficacy, general health status, improvement, satisfaction, work loss, and medication use.

We will also determine the relative efficacy of the two interventions in terms of objective outcomes, lumbar dynamic motion and torso muscle endurance, measured by examiners blinded to treatment group assignment.

## Methods and Design

This study is being conducted at the Wolfe-Harris Center for Clinical Studies at Northwestern Health Sciences University (NWHSU) in Minneapolis, Minnesota. Recruitment began in April 2007 and is now complete. Approval was granted by the Institutional Review Boards of NWHSU and the Minneapolis Medical Research Foundation. Written informed consent was obtained from all participants.

### Study population

Two hundred participants were recruited from the Twin Cities metropolitan area through targeted postcard mailings, brochures at community events, advertisements in online local newspapers, and links on the University's website.

### Inclusion Criteria

Individuals must be 18 years or older with a current episode of LBP at least 6 weeks in duration. Participants' low back complaint must meet the Quebec Task Force classifications of 1, 2, 3, or 4 (individuals with back pain, stiffness, or tenderness with or without musculoskeletal and neurological signs)[20]. The pain must be mechanical in nature: i.e., there is no specific, identifiable etiology but pain can be reproduced by movement or provocation testing. Medications that could affect back pain must be stable for the previous 30 days.

### Exclusion Criteria

Participants are excluded if they have baseline pain scores less than 3 on the 0-10 numerical rating scale, inflammatory or destructive tissue changes of the spine, surgical lumbar fusion or multiple lumbar surgeries, or progressive neurological deficits.

Also exclusionary are ongoing LBP treatments by non-study providers, current or pending litigation, pregnancy or nursing, and certain medical conditions such as blood clotting disorders or severe osteoporosis.

Contraindications to spinal manipulation results in exclusion, as chiropractic care alone makes up the monodisciplinary arm of the study. Subjects, however, may have contraindications to a treatment modality in the multidisciplinary treatment arm (e.g., have a needle phobia) and still be included. In such instances there are multiple other treatment modalities which can be used.

### Eligibility Determination

Interested individuals contact study staff, who administer a short questionnaire by phone to ascertain obvious inclusion/exclusion criteria. Suitable candidates are scheduled for a first baseline evaluation (BEV1), which includes informed consent, a self-report questionnaire, health history, physical examination, and x-rays if indicated.

At completion of the BEV1, a standardized patient profile is created which provides a comprehensive summary of the participant's LBP complaint from a bio-psycho-social perspective. Each profile includes clinical and demographic characteristics: self-report of back pain symptoms, disability, general health status, fear avoidance and self-efficacy measures, and patient perspectives (previous experience with LBP treatments, preferences for care, and expectations of study treatments), as well as physical exam and objective test findings.

The profile is reviewed by a multidisciplinary team of chiropractic and allopathic physicians, radiologists, and project managers during weekly case review meetings. Case review occurs to facilitate consistent interpretation and application of predefined eligibility criteria and is held prior to randomization. Decisions regarding eligibility are documented electronically. If eligible, potential subjects are scheduled for a second baseline visit.

At the second baseline evaluation (BEV2), potential participants undergo a review of informed consent, a self-report questionnaire, and objective biomechanical measures including lumbar dynamic motion and trunk endurance. At the end of this visit, eligible and willing participants are randomly assigned to one of two interventions.

### Randomization

Restricted randomization using a 1:1 allocation ratio has been applied utilizing randomly permuted block sizes. The randomization scheme and block sizes are concealed to ensure blinding of the study team to treatment assignments. As individuals become eligible, sequentially numbered opaque envelopes are drawn to assign treatment.

### Treatment Overview

Patients receive 12 weeks of care in one of two treatment arms: 1) monodisciplinary chiropractic care or 2) multidisciplinary integrative care. Patients are requested not to seek additional care for back pain during the intervention phase; additional health care visits are captured on the self-report questionnaires. All activities related to study treatment are documented using standardized electronic treatment records. Providers also query the patient at each visit regarding additional health care visits and side effects; these are documented in the treatment record.

#### Patient Self Assessment Form

To assess their response to treatment during the intervention phase, patients are asked to complete a Patient Self Assessment Form (PSAF) at the first and subsequent visits. Patients choose a symptom and an activity they identify as most affected by their LBP, rating each on an 11-point scale (from "as good as it could be" to "as bad as it could be"). The PSAF was modified from the Measure Yourself Medical Outcome Profile (MYMOP),[21] a valid, patient-generated measure used to quantify treatment effects important to individual patients. Individual responses are graphed over the course of the intervention period.

#### Care Pathways

During the 12 weeks of active treatment, treating clinicians review patient progress by comparing the PSAF, the self-rated symptom and activity, against benchmarks of expected improvement based upon previous work by study investigators. If progress is not satisfactory, a patient's profile may be returned to the clinical care team for discussion and consideration of changing the treatment plan within the protocols of the respective treatment arm.

Guiding principles for treatment include minimizing fear and catastrophizing, decreasing dependency, being mindful of cost-effectiveness, consideration of patient preferences, and avoiding arbitrary limits upon care.

#### Training

All clinicians underwent training prior to treating patients in the study. Training included information on the background and traditions of each healthcare discipline, reviewing the available clinical evidence on the effectiveness of each modality when used to treat low back pain, applying an evidence-informed practice model, and methods in coming to consensus as a team. For the purpose of this study, an evidence-informed practice model was defined as the combination of the research evidence, clinical experience, and the preferences of the patient. Clinicians were also trained in all the study protocols.

#### Monodisciplinary Chiropractic Care

The monodisciplinary chiropractic care team is made up of three chiropractors who meet weekly to review the profiles of newly randomized patients and to reach consensus on possible treatment options. At the first visit, a consultation is held between the treating chiropractor and patient to establish and agree upon a 12-week care plan.

The chiropractors must be experienced and licensed. They are allowed to utilize any non-proprietary treatment under their scope of practice not shown to be ineffective or harmful. Typical visits last 15-30 minutes. Therapies for which there is supporting evidence are encouraged, including manual spinal manipulation (i.e., high velocity, low amplitude thrust techniques, with or without the assistance of a drop table) and mobilization (i.e., low velocity, low amplitude thrust techniques, with or without the assistance of a flexion-distraction table). Chiropractors may also use hot and cold packs, soft tissue massage, teach and supervise exercise, and distribute the exercise and self-care education materials used in the multidisciplinary treatment arm (described below). The number and frequency of treatment visits is determined by the clinician observing the patient's response to care over time, guided by changes in the self-selected symptom and activity rating on the PSAF.

#### Multidisciplinary Integrative Care

The multidisciplinary integrative care team is made up of 2 chiropractors, 2 massage therapists, 2 traditional Chinese medicine (TCM) practitioners, 2 psychologists, an allopathic physician, and 2 exercise therapists. The team works in a non-hierarchical fashion and all members have an equal voice. Weekly meetings are held to review profiles of newly randomized patients. Discussions are held to develop one or more treatment plans for each patient. Each treatment plan consists of one or more modality represented by the multidisciplinary care team and consensus must be reached among the care team for the plan to be included in the subsequent care consultation with the patient.

During the care consultation, the treatment plan options and rationales developed by the team are presented. The patient then exerts a preference, selecting one treatment plan, and begins 12 weeks of active treatment. The number and frequency of treatment visits for each modality is determined by the provider, guided by changes in the patient's self-selected symptom and activity rating on the PSAF.

The therapy modalities in the multidisciplinary treatment arm can be delivered independently or in combination with one another.

##### Chiropractic

As in the monodisciplinary treatment arm, experienced, licensed chiropractors providing care in the multidisciplinary arm may use any non-proprietary treatment under their scope of practice not shown to be ineffective or harmful. Typical visits last 15-30 minutes. Therapies for which there is supporting evidence are encouraged, including manual spinal manipulation (i.e., high velocity, low amplitude thrust techniques, with or without the assistance of a drop table) and mobilization (i.e., low velocity, low amplitude thrust techniques, with or without the assistance of a flexion-distraction table)[22,23].

##### Cognitive Behavioral Therapy

Cognitive behavioral therapy is provided by licensed psychologists. Sessions last approximately 60 minutes. Treatment focuses on the psychological and social influences affecting LBP and attempts to modify the environmental and cognitive processes contributing to the LBP experience. Because no one technique has demonstrated more effectiveness over another, a variety of operant and respondent cognitive treatment approaches are used[24,25].

##### Exercise

Exercise therapy is provided one-on-one by exercise therapists under the supervision of licensed clinicians. Typical sessions last 40-60 minutes. Patients are shown exercises designed to enhance mobility, coordination, and to increase trunk endurance. These may include flexion/extension motion cycles, hip/knee stretches, prone press ups (back extension), squats, abdominal curl ups, side bridge variations, and leg and arm extension variations[26]. Depending upon their ability and progress, patients may attend a series of supervised exercise therapy sessions or be provided with exercises to perform at home with in-person follow up. Written materials with photos and simple instructions for the exercises are given to the patient.

##### Massage therapy

Therapeutic massage is provided by nationally certified and locally licensed therapists. Sessions last 60 minutes. Massage techniques considered standard practice and not contraindicated in the literature are used[27]. These focus on manual contact for the treatment of soft tissues (i.e., muscle and fascia) and may include neuromuscular therapy, myofascial techniques, trigger point therapy, and classic western style Swedish massage.

##### Medication

Medications are prescribed by a licensed, board-certified medical doctor, with a typical appointment lasting 15-30 minutes. Therapies for which there is supporting evidence and carrying minimal risk are encouraged[28,29]. Choices are individualized, guided by the patient's health history, present medication use, and the presence or absence of contraindications. Medications may include non-steroidal anti-inflammatory drugs (NSAIDS), analgesics, and/or muscle relaxants.

##### Self-care education

Self-care education is provided by exercise therapists under the supervision of licensed clinicians. Typical sessions last 40-60 minutes. Patients are taught spine posture awareness for activities of daily living specific to their abilities, such as lifting, pushing and pulling, sitting and getting out of bed[30,31]. Patients also receive a booklet[32] that encourages movement, the use of ice or heat when needed, and restoration of normal activities.

##### Traditional Chinese Medicine (TCM)

Traditional Chinese Medicine, including acupuncture, is provided by experienced, licensed providers who are National Certification Commission for Acupuncture and Oriental Medicine (NCCAOM) and Clean Needle Technique (CNT) certified. Typical appointments last 45-60 minutes. Clinicians may use any treatment under their scope of practice not shown to be ineffectual or cause harm with the exception of herbs (excluded due to the difficulty in guaranteeing quality) and moxabustion (excluded due to a lack of ventilation in the treatment rooms). Therapies for which there is supporting evidence and carrying minimal risks are encouraged[33,34]. Acupuncture, liquid moxa with a heat lamp, Tui Na (Asian bodywork), and cupping are all permitted.

## Outcome Measures

**Figure 1:**
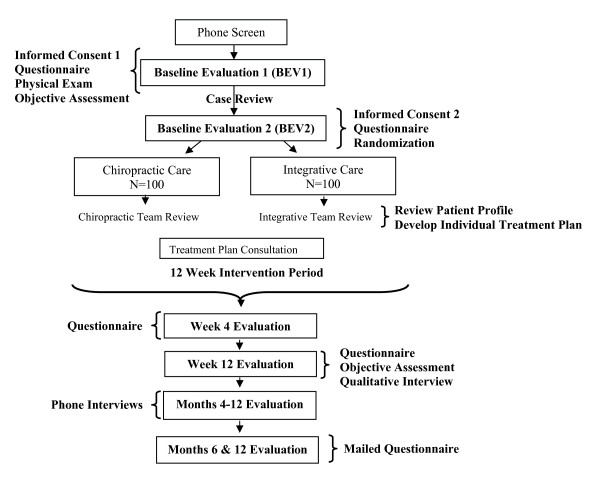
**Participant Flow, Study Visits and Evaluations**. Questionnaires: patient-rated outcomes, expectations, preferences, cost and utility measures. Objective Assessment includes blinded testing of motion and endurance. Phone Interviews include cost-measures. Qualitative Interviews include questions regarding perceptions of treatment, determinants of satisfaction.

Outcomes are measured by patient self-report, blinded objective assessment, and in-person and telephone interviews. Data are collected at baseline, during the 12-week treatment period, and over the course of one year following randomization. Participant flow, study visits, and evaluations are shown in Figure 1.

### Self-report outcome measures

**Patient-rated pain **is the primary outcome measure. Patients are asked to rate their typical LBP over the last week on an ordinal 11-box scale (0 = no LBP, 10 = the worst LBP possible)[35].

Secondary outcome measures include frequency of symptoms, low back disability, fear avoidance, self-efficacy, general health status, improvement, satisfaction, work loss, and medication use.

**Frequency of symptoms **is rated for 1) back pain, 2) leg pain, 3) numbness or tingling in the leg, foot or groin, and 4) weakness in leg or foot on a 0 to 6 scale (0 = not at all, 6 = always)[36,37].

**Low back disability **is measured with the Modified Roland Scale, a 23-item questionnaire that assesses the degree to which LBP restricts daily activities[36,38].

**Fear avoidance **is evaluated with the Fear-Avoidance Beliefs Questionnaire, consisting of 21 items that quantify fear-avoidance beliefs about work and physical activity. Each item is a statement; the patient is asked to rate their degree of agreement/disagreement on a 0 to 6 scale (0 = completely disagree, 6 = completely agree)[39].

**Self-efficacy **is measured by the Pain Self-Efficacy Questionnaire, a 10-item scale used to assess the level of self-confidence in performing functional and social activities despite the presence of pain. Scores range between 0 (no self-efficacy) and 60 (highest self-efficacy)[40].

**General health status **is assessed by the EuroQol 5D, a multi-attribute utility scale (MAUS) covering five dimensions (mobility, self-care, usual activities, pain/discomfort, and anxiety/depression) with three levels (no problem, moderate problem, severe problem)[41,42].

**Improvement **is measured by asking patients to compare their LBP condition to what it was before treatment using a 9-point ordinal scale. Response choices range from no symptoms (100% improvement) to twice as bad (100% worse)[43].

**Satisfaction **is assessed using seven questions addressing different aspects of patient care with 5 response choices (1 = poor, 5 = excellent)[32,44]. Overall satisfaction with care is also measured on a 7-point ordinal scale (1 = completely satisfied, 7 = completely dissatisfied).

**Work Loss **is assessed by three questions based on items from the National Health Interview Survey (NHIS)[45]. Each question asks the patient to reflect back over a specified time period and report the number of days they missed work or school, spent in bed, and cut down on usual activities for more than half a day due to back pain.

**Medication Use**, including non-prescription and prescription medication, is measured using a 5-point scale. Patients indicate how frequently they have taken medication for their LBP in the past week (1 = have not taken any, 5 = taken daily)[44,46].

### Objective outcome measures

Secondary objective outcomes, lumbar dynamic motion and torso muscle endurance, are measured by examiners blinded to treatment assignment at baseline and week 12 (post-intervention).

**Lumbar dynamic motion **is assessed using the Zebris CMS-HS Spine Motion Analyzer (Zebris Inc., Isny im Allgau, Germany)[47,48]. The system acquires 3-dimensional motion data through measurement of ultrasonic pulses traveling between ultrasound emitter arrays applied to the patient and a stable microphone. Patients are asked to perform flexion-extension, rotation, side-bending, and circumduction motion.

**Torso muscle endurance **of the trunk flexors, lateral flexors, and extensors is measured using procedures described by McGill[49]. These tests have been shown to be valid and reliable measures of torso muscle endurance[50,51]. Test data consists of the time (in seconds) that each posture is held. Strength of the trunk flexor and extensor musculature is measured by assessing peak effort in pounds.

### Qualitative measures

Qualitative interviews are conducted with patients individually at the end of the 12-week treatment period[52]. A schedule of questions is used to direct the interviews and keep them on a path consistent with the purpose of the study[53]. The format is semi-structured, with open-ended questions followed by probing questions, if indicated. Patients are asked how they felt about the treatment they received, whether it met their expectations, and what they liked and disliked about treatment. They are also asked to identify factors considered when determining their satisfaction with care. Providers are interviewed when the study is completed. These interviews explore the clinicians' experiences working with other providers in their respective mono- and multidisciplinary clinical care teams, as well as the perceived usefulness of the care pathways.

Permission is sought to audio-record the interviews, and patients are assured confidentiality, allowing them to speak freely in response to the questions[52]. All interviews are transcribed for analyses. To ensure consistency over the course of the study, audio-taped interviews are monitored for standardized interview techniques and 10% of interview transcripts are randomly sampled and compared to recorded interviews for accuracy.

### Cost-Effectiveness and Utility Measures

A societal perspective will be taken as the basis for the cost-effectiveness measurement and analyses. Thus, direct health care costs, direct non-health care costs, and indirect costs will be used as the economic indicators[54,55]. These cost measures are collected from standardized study provider forms at each study treatment visit and patient self-report questionnaires at months 4, 12, 26, and 52. Telephone interviews occur monthly from weeks 12 to 52.

Direct costs for each patient will represent the pain- or disease-related medical costs based on utilization and estimated costs. This will include the costs of the study treatments to which patients are randomized and costs for additional visits to non-study health care providers, prescription medications, advanced imaging (CT, MRI), and hospitalization. Direct non-health care costs will include out-of-pocket expenses (non-prescription medications), informal care, and travel expenses.

Indirect costs will include loss of productivity due to back-related absence from work or days of inactivity. Days in which activity is restricted due to LBP are assessed by three questions based on items from the National Health Interview Survey (NHIS)[45].

Cost utility will be measured using the EuroQol 5D, which has been valued by a large sample of the general population using time trade-off evaluations. It is a brief, easily completed instrument and is considered to be one of the best preference-based measures currently available[41,56].

### Other Measures

Patient expectations are evaluated based on questions used in previous studies by the investigators. Prior to randomization (at BL2), patients are asked to rate how helpful they believe each treatment to be on an 11-box scale (0 = not at all helpful, 10 = extremely helpful)[57].

Patients are asked to report side-effects in the patient self-report questionnaires by choosing from a list of side-effects generated from previous studies by the investigators assessing chiropractic care and exercise[58,59]. For each side-effect chosen, the patient is asked to rate the bothersomeness of the side-effect on an 11-box scale (0 = not at all bothersome, 10 = extremely bothersome). This method of recording side-effects is an attempt to standardize side-effect reporting in clinical trials, which has been inadequately addressed in much of the research performed to date[9].

## Data Analysis

### Treatment Effectiveness

Power was calculated to detect an 8 to 9 percentage point difference between groups in the primary outcome measure of patient self-reported pain after 12 weeks and one year. A sample size of 100 patients per group provides 80% power assuming an alpha level of .05. This allows for a 15% loss to follow up.

Mixed-Model analysis of covariance (ANCOVA) will be used to test for differences between groups in patient-rated primary and secondary outcomes in both the short- (12 weeks) and long-term (52 weeks). Baseline values will be used as covariates when appropriate; intention-to-treat analysis will be used[60]. A sensitivity analysis will be performed, including patient expectations as a covariate to assess its influence on study results.

### Cost-effectiveness Analysis

A cost comparison of the intervention groups using data on direct and indirect costs will be conducted. Cost differences between groups will be estimated for all costs up to weeks 12, 26, and 52. ANCOVA will be used as in the primary analysis to yield the mean difference between groups, adjusted for baseline costs and other important baseline covariates that are identified.

A cost-effectiveness analysis (CEA) comparing the two intervention groups using pain as the effectiveness measure will also be performed. Cost-effectiveness will be evaluated with the incremental cost-effectiveness ratio (ICER). The ICER is defined for this study as the adjusted mean difference between groups in costs divided by the adjusted mean difference in patient-rated pain. ICER will be calculated at the same time points used in the primary analysis.

A cost-utility analysis (CUA) will be performed to compare the intervention and control groups using the EuroQol-5D. Utility will replace pain in the ICER.

### Qualitative Analysis

Content analysis using an inductive approach[61] will be used to identify categories and themes that occur in the transcribed text generated from the qualitative interviews[62]. Transcribed data will be entered into a database (ATLAS.ti) designed to capture and analyze qualitative data.

The text of ten interviews will be read independently by two investigators to gain an overall impression and to establish and define preliminary codes in response to the proposed questions[63,64]. After the initial analysis, the investigators will meet to reach a consensus on preliminary codes. These codes will be entered into a "code book," which will provide a detailed definition for each code[62]. Information related to methodological decisions and their rationale will also be documented[61]. Subsequent transcripts will be independently reviewed to identify and code text segments and to assess the inter-rater reliability of text coding. Kappa values of less than 0.8 will necessitate review of the coding structure.

Investigators will meet after each set of twenty interviews to revise the code book and add new codes as necessary, grouping them into thematic categories. The frequency of themes will be quantified and representative quotations will be identified[62,65]. The investigators will independently review the summarized information and verify it for consistency with the original text. Categorized information from the transcribed interviews will be entered in matrices to organize and display categories by treatment groups as a means of illustrating relationships among categories[66]. This information will then be summarized and interpreted. Findings will be discussed until consensus is reached. The frequency of themes will be quantified and representative quotations will be identified[62,65]. The frequency of responses in the thematic categories will be cross-tabulated with treatment group assignment and compared for between-group differences using Chi-square analysis. 95% confidence intervals will be calculated for these differences.

## Discussion

The design of this randomized clinical trial (RCT), with cost-effectiveness and qualitative studies conducted alongside the RCT is innovative in several ways.

First, it uses flexible clinical care pathways, which allow for provider and patient choice within the context of study protocols. The ability of the clinician to assess change in the patient's self-selected symptoms mimics real-world care, while also valuing the provider's clinical experience. It also forces, in the multidisciplinary treatment arm, discussion across disciplines; when patients don't meet benchmarks, their case is returned to the care team for review and possibly a change in treatment plan. It has been suggested that combining modalities could have a synergistic effect that might lead to greater improvements;[67] this trial encourages the combination of CAM and conventional treatment. Finally, allowing patients to express their treatment plan preference may increase positive treatment effects. Although this study cannot examine preference alone, patients' qualitative experiences will be captured, which can lead to greater understanding of how preference may influence treatment outcomes.

Second, the multidisciplinary intervention is a non-hierarchical model where consensus is reached among the conventional medicine and CAM team members when formulating treatment plans. The advanced level of integration of the multidisciplinary team, where disparate practitioners reach decisions and consensus as a group, will inform future endeavors by modeling one possible method of practice.

Finally, this study applies the best available evidence for both conventional medicine and CAM to meet the needs of individual LBP patients. Individualizing care has the potential to maximize outcomes in non-specific low back pain when multiple efficacious treatments by themselves have modest effects when applied to the group overall. This study may add to our understanding of the biopsychosocial model of low back pain and whether it could be translated into a useful profile of each patient to guide care.

The study is anticipated to be completed in 2010, at which time results will be made available.

## Competing interests

The authors declare that they have no competing interests.

## Authors' contributions

The authors are investigators of the study and each has contributed to the study design. GB, MM, and RE are responsible for the conceptualization of the trial. GB is the principal investigator of the HRSA grant award. GB and MM are co-lead investigators for project implementation. MM and KW are responsible for trial coordination. KW prepared the first draft of the manuscript and organized revisions. All authors read and approved the final manuscript.
